# Sociodemographic profiles, educational attainment and physical activity associated with The Daily Mile™ registration in primary schools in England: a national cross-sectional linkage study

**DOI:** 10.1136/jech-2020-214203

**Published:** 2020-10-01

**Authors:** Tishya Venkatraman, Kate Honeyford, Céire E Costelloe, Ram Bina, Esther M F van Sluijs, Russell M Viner, Sonia Saxena

**Affiliations:** 1 Primary Care and Public Health, Imperial College London, London, UK; 2 MRC Epidemiology Unit, University of Cambridge, Cambridge, UK; 3 UKCRC Centre for Diet and Activity Research (CEDAR), University of Cambridge, Cambridge, UK; 4 Population, Policy and Practice Research Programme, UCL Institute of Child Health, London, UK

**Keywords:** Physical activity, public health policy, obesity, epidemiology

## Abstract

**Objective:**

To examine primary school and local authority characteristics associated with registration for The Daily Mile (TDM), an active mile initiative aimed at increasing physical activity in children.

**Design:**

A cross-sectional linkage study using routinely collected data.

**Setting:**

All state-funded primary schools in England from 2012 to 2018 (n=15,815).

**Results:**

3,502 of all 15,815 (22.1%) state-funded primary schools in England were registered to do TDM, ranging from 16% in the East Midlands region to 31% in Inner London. Primary schools registered for TDM had larger mean pupil numbers compared with schools that had not registered (300 vs 269, respectively). There was a higher proportion of TDM-registered schools in urban areas compared with non-urban areas. There was local authority variation in the likelihood of school registration (intraclass correlation coefficient: 0.094). After adjusting for school and local authority characteristics, schools located in a major urban conurbation (OR 1.46 (95% CI 1.24 to 1.71) urban vs rural) and schools with a higher proportion of disadvantaged pupils had higher odds of being registered for TDM (OR 1.16 (95% CI 1.02 to 1.33)). Area-based physical activity and schools’ educational attainment were not significantly associated with registration to TDM.

**Conclusion:**

One in five primary schools in England has registered for TDM since 2012. TDM appears to be a wide-reaching school-based physical activity intervention that is reaching more disadvantaged primary school populations in urban areas where obesity prevalence is highest. TDM-registered schools include those with both high and low educational attainment and are in areas with high and low physical activity.

## INTRODUCTION

The WHO and the UK government recommend that children aged 5 to 17 years should accumulate at least 60 minutes of moderate to vigorous physical activity (MVPA) daily.^1 2^ However, only one in six children and young people in the UK meet this recommendation and less than two-thirds achieve 30 min of MVPA a day.^3^ Schools are considered ideal settings for children to meet most of their physical activity requirements since they spend a large proportion of their time at school. There is potential for all children to take part, especially those who tend to be less physically active than their peers, such as girls, those residing in deprived areas and some minority ethnic groups.^3^

The Daily Mile (TDM) is an active mile initiative that began in Scotland in 2012 and has grown as an organic grass-roots movement. TDM has reportedly had a large uptake that has spread across the UK and Europe, with an estimated 10,000 schools and nurseries taking part across 77 countries worldwide.^4^ It is a teacher-led activity that involves primary school children jogging or running for 15 min during curriculum time within the school grounds.^5^ Its core features are that it is simple, inclusive and has flexible delivery that can be adapted to different primary school settings.^5^ Since 2018, the UK’s Child Obesity Strategy specifically mentioned TDM in outlining a national ambition for every primary school to achieve physical activity targets.^6^ Since then, there have been considerable efforts to promote and implement TDM. This includes 1.5 million pounds of investment by the UK government into funding co-ordinators in 10 areas of England to encourage schools to register for TDM.^7 8^ TDM Foundation has run numerous campaigns to promote TDM such as TV advertisements and a running event for primary school children called ‘GORunforFun’ which engaged 2,400 children from 45 schools across London.^9 10^ Qualitative research suggests there is considerable adaptation that occurs in areas where TDM has been successfully implemented.^11^ These features could be a key to its success in implementation and explain why more complex school-based interventions have failed.^12 13^ A growing evidence base suggests that TDM increases physical activity and fitness over the first 12 months of its adoption,^14 15^ but evidence of its impact on educational performance, well-being or maintaining healthy weight is limited.^16 17^

Many schools, particularly those in urban areas, have limited access to outdoor space or have concerns about the risks of exposing children to air or traffic pollution, which could act as disincentives for uptake of TDM and other active mile interventions that involve running or jogging outside. Key performance indicators of educational attainment in UK schools are firmly tied to performance in reading and writing scores. Thus, it is possible that primary schools that lag in performance league tables do not prioritise activities such as TDM that may compete with curricular time. The purpose of this study was to link multiple routine data sources to examine sociodemographic, health and educational profiles of primary schools registered for TDM and the local authority areas in which they are located in England.

## METHODS

### Design

This was a cross-sectional study of all state-funded primary schools in England. We created a database linking health and education data from the School Census, the National Pupil Database, Active Lives Surveys and the National Child Measurement Programme, with TDM registration data provided by TDM Foundation.

### Data sources

The School Census collects aggregate information annually from all state-funded schools and includes data on more than 15,000 schools and over 4.5 million children.^18^ It holds data on pupil characteristics including pupil numbers, ethnicity, if they are disadvantaged, or whether they have any special educational needs (table 1). The National Pupil Database includes pooled data, based on multiple data collections on pupils aged 3 to 19 years in state-funded schools in England. It contains data on pupils’ educational attainment from national annual standardised assessments conducted each academic year.^19^ It also includes data on pupils’ absences.

**Table 1:** List of variables included in the model

Variables for models	Data source	Definition
**School variables**		
Pupil number	School Census	Number of boys and girls enrolled in the school according to the school census
School type	School Census	Local authority-controlled or academy
Rurality	School Census	Office for National Statistics Classification: Rural: hamlets and isolated dwellings, town and fringe, and village Urban: urban city and town, major conurbation and minor conurbation
Percent of disadvantaged pupils	School Census	Eligible for free school meals or have been in the last 6 years; looked after children, or those who have previously looked after by the state, but are now adopted or are subject to a special guardianship order, a child arrangements order, or a residence order; children with parents in the armed forces
Percent of pupils whose first language is known or believed to be other than English	School Census	A proxy measure for ethnic diversity: a pupil’s first language is defined as any language other than English that a child was exposed to during early development and continues to be exposed to at home or in community
Educational attainment—percent of pupils reaching the expected standard in reading, writing and maths	National Pupil Database	Educational attainment defined as the expected standard in reading, writing and mathematics. It is a scaled score of 100 or above that is derived from standardised testing. A scaled score of 100 or more signifies a child is working at the expected national standard, while a score below 100 indicates that a child has not reached the government expected national standard. The maximum score possible is 120, and the minimum is 80
**Local authority variables**		
Adult physical activity levels	Active Lives Adults Survey	Percent of adults reporting they are doing moderate or vigorous activity for more than 150 min a week
Child physical activity levels	Active Lives Children and Young People Survey	Percent of children reporting they are doing moderate or vigorous activity for 30 min or more of both at school and outside school every day
Adult excess weight status prevalence	Active Lives Adults Survey	Percent of adults (aged 18+) classified as overweight or obese
Child excess weight status prevalence	National Child Measurement Programme	Percent of children classified as overweight or obese at reception, the beginning of primary school when children are 5–6 years old

### School populations (table 1)

Our population was all state-funded primary schools in England, including academies and free schools which report directly to the central government, and local authority-controlled schools which report to local government.

We included all primary schools in the 2018 School Census that had a valid Unique Reference Number (supplemental figure 1). We excluded secondary schools, nurseries and day care centres and removed schools that were closed on the date of data collection, for example, those that had converted to an academy, to avoid duplication. In order to minimise loss of observations due to missing data in a single year, we used the mean of available data from up to 6 years from the School Census and the Absence Extract of the National Pupil Database, and from up to 3 years of educational attainment data from the National Pupil Database.^19 20^ Thereafter, we used a complete case analysis to run the models. We conducted a sensitivity analysis to explore differences in characteristics between schools included and excluded from the models (supplemental table 1).

10.1136/jech-2020-214203.supp2 Supplementary data

10.1136/jech-2020-214203.supp1 Supplementary data

Within our primary schools’ database, we defined TDM registration as all primary schools that were officially registered with TDM Foundation on August 1, 2019. We assumed that schools that were not officially registered with TDM Foundation were not taking part in TDM.

### Local authority profiles

There are a total of 152 counties and unitary authorities in England which include metropolitan districts, London boroughs, unitary authorities and county councils which were defined as local authorities for our study.^21^ The National Child Measurement Programme is a nationally mandated surveillance programme that collects height and weight converted to weight status for over 1 million children (98% of all children) entering and leaving primary school in England each year.^22^ The Active Lives Adult Survey collects data on physical activity and reported weight status of adults, and the Active Lives Children and Young People Survey contains reported data on children’s physical activity in England.^23^ We linked population profiles of obesity prevalence and physical activity in each local authority to our schools’ database.

### Statistical analysis

We compared school and local authority characteristics of primary schools registered for TDM with those that did not register. We also examined the local authority health profiles of child and adult physical activity and obesity prevalence in areas in which primary schools were located. We used t-tests to compare continuous variables (eg, % white pupils) and ᵡ² tests to compare categorical variables (eg, counts of schools by region).

Due to the hierarchical nature of the data, multilevel models were used to determine the association between school and local authority characteristics associated with TDM registration and to assess variation at school and local authority levels. To investigate the variation explained at different levels, a sequential series of models were built (box 1).

**Box 1 Model building and assessment**

**Model building**

- In order to establish the need to account for clustering, we fitted logistic regression models with school and local authority characteristics (supplemental table 2).
- After checking the need to account for clustering, we fitted a multilevel model with intercept only to examine the variability of TDM registration between local authorities (model 1). Next, to test whether school characteristics explained variation in registration, we extended model 1, adding school-level characteristics (model 2). Finally, we extended model 2 adding local authority characteristics (adult and child excess weight status and physical activity) (model 3) to determine the extent of variation.

**Model comparison**

- Models 1, 2 and 3 were run as logistic regressions, without the local authority–level random effect. This was in order to obtain a log-likelihood ratio χ^2^ test, to test the multilevel logistic regression model against the corresponding non-hierarchical logistic regression model to assess the goodness-of-fit. Results of the log-likelihood ratio χ^2^ test, along with the Akaike Information Criterion values, showed that multilevel models were preferred. Model 3 was chosen as the best fit for the data.

10.1136/jech-2020-214203.supp3 Supplementary data

To examine primary school and local authority characteristics associated with registration for TDM, we selected 10 candidate variables that are proxy indicators of school and pupil health and well-being. These were identified from the literature and by consensus among authors (table 1). These variables were then included in regression models. As the numeric values of the variables of interest in our models were on different scales of magnitude, we scaled all the continuous variables for the multilevel models through division by the SD for the analyses $\left(\zeta =\frac{x-\mu }{\sigma }\right)$. All school and local authority characteristics were included in the final model (model 3), which assessed the association between school and local authority characteristics with TDM registration. For all the models, variables with an alpha level of <0.05 were considered statistically significant. All statistical analysis was done using R software version 3.5.2 (December 20, 2018).

### Patient and public involvement

This study design and background were informed by extensive knowledge exchange with health professional, educational stakeholder representatives including head teachers, parents, policy-makers, TDM Foundation, and experts and academics including TDM Research Advisory Group.

## RESULTS

### School characteristics, educational attainment and local authority population health profiles

Between 2012 and 2018, there were 15,815 state-funded primary schools in England (table 2). Of these, 3,502 schools (22.14%) were registered for TDM. School populations ranged from 7 to 1431 pupils (mean 275), and 30% of pupils were classed as disadvantaged overall.

**Table 2:** Sociodemographic characteristics of primary schools in England by The Daily Mile (TDM) registration (level 1) (n=15,815)

	All primary schools mean (SD)	TDM-registered schools mean (SD)	Non-TDM-registered schools mean (SD)	P value	Schools reporting (N)
School size (number of pupils)	275.92 (156.72)	299.79 (157.41)	269.13 (155.87)	<0.001*	15815
Disadvantaged pupils (%)	30.06 (19.07)	31.87 (19.46)	29.52 (18.92)	<0.001*	13585
% Ethnic group					
White British and White Other	79.29 (24.57)	76.00 (26.64)	80.2 (23.86)	<0.001*	15815
Asian	7.94 (15.52)	9.54 (17.47)	7.48 (14.88)	<0.001*	15815
Black African and Black Caribbean	4.35 (9.54)	5.32 (10.77)	4.07 (9.13)	<0.001*	15815
Mixed	5.21 (4.17)	5.58 (4.32)	5.10 (4.12)	<0.001*	15815
Other	2.6 (4.33)	2.89 (4.50)	2.52 (4.28)	<0.001*	15815
Unclassified	0.77(1.64)	0.78 (1.53)	0.77 (1.66)	0.602*	15743
Pupils known to be eligible for and claiming free school meals (%)	14.06 (11.19)	15.1 (11.52)	13.8 (11.10)	<0.001*	15743
Absence rate (%)	4.10 (0.87)	4.13 (0.86)	4.10 (0.87)	0.042*	15815
Pupils whose first language is known or believed to be other than English (%)	15.51(21.34)	17.97 (22.85)	14.81 (20.84)	<0.001*	15743
SEN pupils with a statement or EHC plan (%)	1.93 (2.68)	1.97 (2.52)	1.92 (2.72)	0.36*	13585
Pupils reaching the expected standard in reading, writing and maths (%)	62.45 (14.67)	62.69 (14.15)	62.38 (14.83)	0.292*	13585
Progress measure for reading, writing and maths	0.13(1.84)	0.21 (1.77)	0.110 (1.86)	<0.05*	13548
	**All primary schools** **N (%)**	**TDM-registered schools** **N (%)**	**Non-TDM--registered schools** **N (%)**	**P value**	
School type					
Academy	4113 (26.01)	927 (28.44)	3186 (8.54)	0.33†	14116
Local authority–controlled	10 003 (63.25)	2332 (71.56)	7671 (21.48)		
Office for National Statistics Rurality Classification					15815
Hamlets and isolated dwellings (rural)	766 (4.84)	134 (3.83)	632 (5.13)	<0.001†	
Town and fringe (rural)	1727 (10.92)	314 (8.97)	1413 (11.48)		
Village (rural)	2072 (13.1)	335 (9.57)	1737 (14.11)		
City and town (urban)	6012 (38.01)	1199 (34.24)	4813 (39.09)		
Major conurbation (urban)	4711 (29.79)	1408 (40.21)	3303 (26.82)		
Minor conurbation (urban)	527 (3.33)	112 (3.2)	415 (3.37)		
Region					15815
North East	1492 (9.43)	203 (5.8)	598 (4.86)	<0.001†	
North West	1839 (11.63)	639 (18.25)	1734 (14.08)		
Yorkshire and the Humber	686 (4.34)	334 (9.54)	1338 (10.87)		
East Midlands	801 (5.06)	247 (7.05)	1245 (10.11)		
East of England	2373 (15)	412 (11.76)	1427 (11.59)		
West Midlands	1061 (6.71)	332 (9.48)	1323 (10.74)		
Inner London	2508 (15.86)	214 (6.11)	472 (3.83)		
Outer London	1728 (10.93)	285 (8.14)	776 (6.30)		
South East	1655 (10.46)	501 (14.31)	2007 (16.30)		
South West	1672 (10.57)	335 (9.57)	1393 (11.31)		

*P value derived from t-test comparing variable and TDM registration.

†P value derived from χ² test comparing variable and TDM registration.

EHC, education, health and care plan; SEN, Special Educational Needs.

Progress measures are a value-added measure that pupils’ results are compared to the actual achievements of other pupils nationally with similar prior attainment. Progress scores will be centred around 0, with most schools within the range of −5 to +5.^24^

TDM schools had greater mean pupil numbers compared to non-TDM schools (300 vs 269, respectively). The proportion of disadvantaged children in TDM schools was higher than non-TDM schools (32% vs 30%, respectively). In line with our sample, which was all state-funded primary schools across England, 72% of TDM schools were under local authority control and 28% were academies (including free schools). A greater proportion of TDM schools were located in urban areas, especially the major conurbations of London, the West Midlands, West Yorkshire, Tyneside, Merseyside and Greater Manchester (table 2). Further, TDM schools had a higher proportion of pupils whose first language was not English (18% vs 15%, respectively), a higher proportion of pupils from black and minority ethnic groups and higher academic progress measures compared with non-TDM schools (0.21 vs 0.11, respectively). The registration of TDM ranged from 16% in the East Midlands region to 31% in Inner London. There are a total of 152 counties and unitary local authorities in England, all of which were in our sample (table 3). Across all local authorities, the mean percentage of children meeting recommended levels of physical activity was 14%, and the proportion of overweight or obese children rose from 1 in 5 (at age 5) to around 1 in 3 (at age 11).

**Table 3:** Characteristics of local authorities included in the study in England (level 2) (n=152)

	Mean (SD)	Local authorities reporting (N)
% of adults who report being physically active for 150 min or more per week	62.21 (4.37)	124
% of children reported as doing moderate or vigorous physical activity for 30 min or more of both at school and outside school every day	13.98 (2.35)	152
% of adult excess weight	65.26 (4.10)	152
% of overweight or obese children reception (aged 5 years)	22.35 (2.54)	150
% of overweight or obese children in year 6 (aged 11 years)	33.66 (4.04)	150

### Characteristics associated with TDM registration

Table 4 presents results of the modelling process. There was significant variation in TDM registration by local authority (intercept only model 1). The estimated local authority level effects from the intraclass correlation coefficient (ICC) of model 1 was 0.094, this infers that ≈9.4% of the total variance in TDM registration by schools is explained by local authority effects.

After adjusting for school-level characteristics (model 2), schools in a major urban conurbation showed almost 50% higher odds of being registered for TDM (OR 1.46 (95% CI 1.24 to 1.71)). Additionally, schools with a higher proportion of disadvantaged pupils had higher odds of being registered as a TDM school compared with schools with a lower proportion of disadvantaged pupils, whereby an increase of 1 SD (19%) in the proportion of disadvantaged pupils produces, on average, a 16% increase in the odds of being a TDM school (unscaled adjusted OR 1.16 (95% CI 1.0 to 1.33)). In the fully adjusted model (model 3), no significant associations were found between registration with TDM and child and adult physical activity, child and adult excess weight status, educational attainment measures and pupils whose first language was not classified as English.

**Table 4:** Multilevel multivariable logistic regression of school and local authority characteristics on The Daily Mile registration in England (level 1, N=12,214 primary schools; level -2, N=124 local authorities) using scaled parameters

		Model 1: intercept only		Model 2: +school variables		Model 3: +local authority variables		
#	Parameters	OR (95% CI)	SE	Scaled OR (95% CI)	SE	Scaled OR (95% CI)	SE	Unscaled OR (95% CI)
1	Local authority–controlled schoolⱡ			1.06 (0.96, 1.17)	0.05	1.07 (0.96, 1.19)	0.05	
2	Hamlets and isolated dwellings (rural)ⱡ			0.95 (0.75, 1.21)	0.12	0.96 (0.76, 1.22)	0.12	
3	Town and fringe (rural)§			1.01 (0.87, 1.19)	0.08	1.03 (0.87, 1.21)	0.08	
4	Village (rural)§			0.88 (0.75, 1.04)	0.08	0.88 (0.74, 1.04)	0.09	
5	Major conurbation (urban)§			1.46‡ (1.24, 1.71)	0.08	1.46‡ (1.22, 1.74)	0.09	
6	Minor conurbation (urban)§			1.04 (0.73, 1.48)	0.18	0.93 (0.63, 1.37)	0.2	
7	% of pupils whose first language is known or believed to be other than English			1.00 (0.95, 1.05)	0.03	1.01 (0.95, 1.07)	0.03	1.23 (0.33, 4.59)
8	% of pupils reaching the expected standard in reading, writing and maths			0.99 (0.95, 1.04)	0.02	1.00 (0.96, 1.05)	0.02	0.98 (0.50, 1.91)
9	% of disadvantaged pupils			1.01† (1.00, 1.02)	0	1.01* (1.00, 1.02)	0.004	1.16 (1.02, 1.33)
10	% of overweight or obese adults					0.97 (0.86, 1.11)	0.07	0.89 (0.51, 1.52)
11	% of physically active adults					0.93 (0.83, 1.05)	0.06	0.73 (0.44, 1.22)
12	% of physically active children					1.02 (0.93, 1.11)	0.04	1.04 (0.85, 1.26)
13	% of children who are overweight or obese					0.97 (0.88, 1.07)	0.05	0.92 (0.72, 1.17)
**Model summary**								
Intercept		0.29‡ (0.26, 0.32)	0.05	0.22‡ (0.18, 0.26)	0.088	0.22‡ (0.18, 0.26)	0.09	
ICC		0.09		0.07		0.06		
Likelihood ratio χ2 test (ordinary logistic vs multilevel logistic model)		557.42‡		302.24‡		247.12‡		
AIC/BIC		16 185.3 (BIC)		14 290.5 (BIC)		12 688.2 (BIC)		
		16 170.0 (AIC)		14 207.8 (AIC)		12 577.0 (AIC)		

*P≤0.05.

†P≤0.01.

‡P≤0.001.

§Reference group=city and town.

ⱡ Reference group = Academy

Parameters from 1 to 13 all are fixed-effects estimates. AIC, Akaike information criterion; BIC, Bayesian information criterion; ICC, intraclass correlation coefficient.

We found that 9.4% of the residual variance in TDM registration by schools is explained by local authority effects and 90.6% is explained by the schools’ characteristics. When school characteristics were added to the intercept only model, only part of the variation observed in model 1(2.6%) was explained (model 2 ICC=0.068). The ICC for model 3 is 0.064, dropping only very marginally from model 2, even after adding local authority prevalence of excess weight status and physical activity variables for children and adults, suggesting that the adult and child physical activity and excess weight status account for only ~0.4% of the variation seen in TDM registration.

## DISCUSSION

### Principal findings

One in five primary schools across England has registered with TDM since 2012. The distribution varies across the 10 English regions, but it is much higher in London and major urban conurbations where over a third of primary schools are registered.

After adjusting for school and local authority characteristics, larger primary schools in urban areas and a higher proportion of disadvantaged children were more likely to have registered for TDM. We found no association between TDM registration and area-based physical activity or excess weight status, or schools’ educational attainment. Additionally, there was evidence of variation in registration of TDM in different local authorities that was not accounted for by characteristics of schools and its pupils.

### Strengths and limitations

To our knowledge, this study is the first national study to characterise primary school uptake of a whole school-based physical activity intervention. Its strengths are its size and use of routine data that is nationally representative of around 4.7 million children in over 15,000 state-funded primary schools and all local authorities in England,^25^ reducing selection bias. The use of multilevel models allowed us to account for the clustering of pupil data within schools, and population health indicators in local authorities, as well as adjust for important confounders.

However, there are a number of important limitations to our study. The models included 12,214 of the 15,815 schools due to missing data. However, the sensitivity analysis (supplemental table 1) shows that there was no selective bias as schools modelled were similar to those excluded. The accuracy of registration data for TDM provided by TDM Foundation, which contains a list of schools registered for TDM via their online official website, was not validated. We consider registration to be an intention to adopt TDM rather than a proxy measure of participation in TDM.^26 27^ We were unable to obtain physical activity for individual children or schools and have instead relied on reported area-based measures of children’s physical activity and is subject to recall bias.

### Findings compared with previous studies

Our findings that more TDM-registered schools were located in major urban conurbations like London could be a result of media campaigns and promotion of TDM and public health endorsement of TDM in some local authorities.^28 29^ There is mixed evidence surrounding the association between children’s physical activity and living in urban or rural areas.^30 31^ Some studies^31 32^ have found that children in urban areas are less physically active compared with rural areas. This is unsurprising, given the multiplicity of ways in which attributes of the physical and built environment affect physical activity in children and adults.^33^ Nevertheless, our findings may be evidence of a successful movement to drive up physical activity among children in urban areas. This is important since 33.5% of the UK population lives in major urban conurbations.^34^ Additionally, we found that after adjusting for rurality and ethnicity, having a higher proportion of disadvantaged pupils in a school increased the odds of adopting TDM. This is inconsistent with previous studies,^35^ which have found that people from lower socioeconomic groups are less likely to be physically active compared with those from higher socioeconomic groups. Further, a recent study of TDM in Wales showed similar benefits in fitness among children from both low and high socioeconomic groups.^15^

Our finding that schools registering for TDM have similar educational attainment to the wider population of schools also suggests it is a wide-reaching intervention that reaches both high-performing and low-performing schools. The evidence base about the effect of school-based physical activity interventions, active miles and TDM itself on cognition and educational attainment is limited.^16 36^ Previous studies of school-based physical activity interventions have not shown they are effective in increasing MVPA in children.^12 13 37^ Possible reasons for this may lie in difficulties implementing complex interventions at scale. Qualitative research suggests there is considerable adaptation that occurs in areas where TDM has been successfully implemented^11^ and may underpin TDM’s success as a growing grass-roots movement.

After adjustment, we found that local authority health profiles of TDM-registered schools were comparable to non-registered schools with respect to child and adult excess weight, and child and adult physical activity. We found no studies examining the impact of local authority on adoption of school-based physical activity interventions in children, but Rind *et al* found significant and distinctive variation in physical activity across local authorities in England in adults.^38^ In England, where almost 65% of state schools are local authority–controlled, local authority commitment to school-based physical activity interventions is vital in helping children reach physical activity recommendations. This lack of association with area-based measures does not preclude a school-level or pupil-level effect.

### Implications for policy and practice

Our findings that TDM is potentially reaching over 1 million children in England are evidence of the appeal to schools. It appears to be a wide-reaching intervention reaching high-performing and low-performing primary schools alike and local authority areas with better and poorer health. If it can be assumed that the physical fitness benefits of TDM seen in small trials to date are sustainable,^14 15 17^ then our findings that it is taken up by urban schools serving disadvantaged school populations suggest added value from TDM over other interventions, that when scaled have failed to reach populations of children most in need.^39^

TDM is promoted as a ‘simple and free’ and ‘sustainable’ intervention.^5^ Our findings support the idea that TDM being ‘simple and free’ might make it a more accessible intervention. If school-based physical activity interventions are embedded in the school curriculum, they are more likely to be effective and sustainable^40^ and TDM could be a vehicle to help children achieve physical activity recommendations. A previous study reporting a process evaluation in the East Midlands region has demonstrated high rates of implementation of TDM during the adoption phase.^41^

A recent study found that TDM increases cardiorespiratory fitness in children from both deprived and affluent backgrounds.^15^ If TDM can improve cardiorespiratory fitness equally and be an accessible solution to reduce the growing inactivity in children, it is important to understand whether TDM is an equitable intervention reaching varied high-risk populations. We recommend further research to explain variation among local authority areas in registration to TDM. There is extensive variation in child obesity prevalence locally and nationally.^42 43^ Since increasing physical activity levels in children is part of the solution for obesity prevention and reduction in children,^6^ it is of interest to understand how inputs from certain local authorities may influence child obesity trends.

## CONCLUSION

One in five primary schools in England has registered for TDM since 2012. TDM appears to be a wide-reaching school-based physical activity intervention that is reaching more disadvantaged primary school populations in urban areas where obesity prevalence is highest. TDM-registered schools include those with both high and low educational attainment and areas with high and low physical activities.

**What is already known on this subject**

- A large proportion of children in the United Kingdom and the world do not meet physical activity recommendations.
- The Daily Mile has been taken up in many countries and has shown to increase physical fitness in children, but there is limited evidence of its distribution within countries or of the characteristics of school populations it is reaching.

**What this study adds**

- More than one in five primary schools in England is registered for The Daily Mile.
- The Daily Mile appears to be a wide-reaching school-based physical activity intervention that is reaching more disadvantaged primary school populations in urban areas where obesity prevalence is highest.
- The Daily Mile registered schools include those with both high and low educational attainment and are located in areas with high and low physical activities.
