# Supplementary material for: Sociodemographic profiles, educational attainment and physical activity associated with The Daily Mile™ registration in primary schools in England: a national cross-sectional linkage study
Source: J Epidemiol Community Health. 2020 Oct 1;75(2):137–44. doi: 10.1136/jech-2020-214203 (PMC7815899; doi:10.1136/jech-2020-214203)
Supplement: Supplementary data [file jech-2020-214203supp002.pdf]

SUPPLEMENTARY INFORMATION

Table S1: Sensitivity Analysis of missing data for variables included in the models

**Parameter**	**Included Primary Schools (N=12214) N(%)**	**Excluded Primary Schools (N=3601) N(%)**
**School Type**		
Academy	3395 (27.80)	718 (19.93)
Local authority controlled	8819 (72.20)	1184 (32.88)
Missing	-	1699 (47.18)
**Office for National Statistics Rurality Classification**		
Hamlets and Isolated Dwellings (Rural)	618 (5.05)	148 (4.10)
Town and Fringe (Rural)	1425 (11.66)	302 (8.39)
Village (Rural)	1774 (14.52)	298 (8.28)
City and Town (Urban)	4776 (39.11)	1236 (34.32)
Major Conurbation (Urban)	3236 (26.49)	1475 (40.96)
Minor Conurbation (Urban)	385 (3.17)	142 (3.95)
Missing	-	-
	**Included Primary Schools (N=12214) mean (SD)**	**Excluded Primary Schools (N=3601) mean (SD)**
**Pupils whose first language is known or believed to be other than English (%)**	14.00 (19.98)	20.73 (24.79)
**Pupils reaching the expected standard in reading, writing, and maths (%)**	62.15 (14.73)	65.12 (13.87)
**Disadvantaged pupils (%)**	28.94 (18.71)	40.07 (19.34)
	**Included local authorities Mean (SD) (N=124)**	**Excluded local authorities Mean (SD) (N=28)**
% Adults who report being physically active for 150 minutes or more per week)	61.84 (5.32)	62.43 (5.77)
% Children reported as doing moderate or vigorous physical activity for 30 minutes or more of both at school and outside school every day	14.02 (2.83)	All missing
% Adult Excess Weight	65.05 (4.62)	62.52 (8.12)
% Overweight or obese children Reception (aged 5 years)	22.51 (2.91)	22.52 (2.83)
% Overweight or obese children in Year 6 (aged 11 years)	34.32 (4.57)	36.82 (3.36)
