# Supplementary material for: Sociodemographic profiles, educational attainment and physical activity associated with The Daily Mile™ registration in primary schools in England: a national cross-sectional linkage study
Source: J Epidemiol Community Health. 2020 Oct 1;75(2):137–44. doi: 10.1136/jech-2020-214203 (PMC7815899; doi:10.1136/jech-2020-214203)

Figure S1: Flow diagram of routine data sources used to create database of primary schools in England

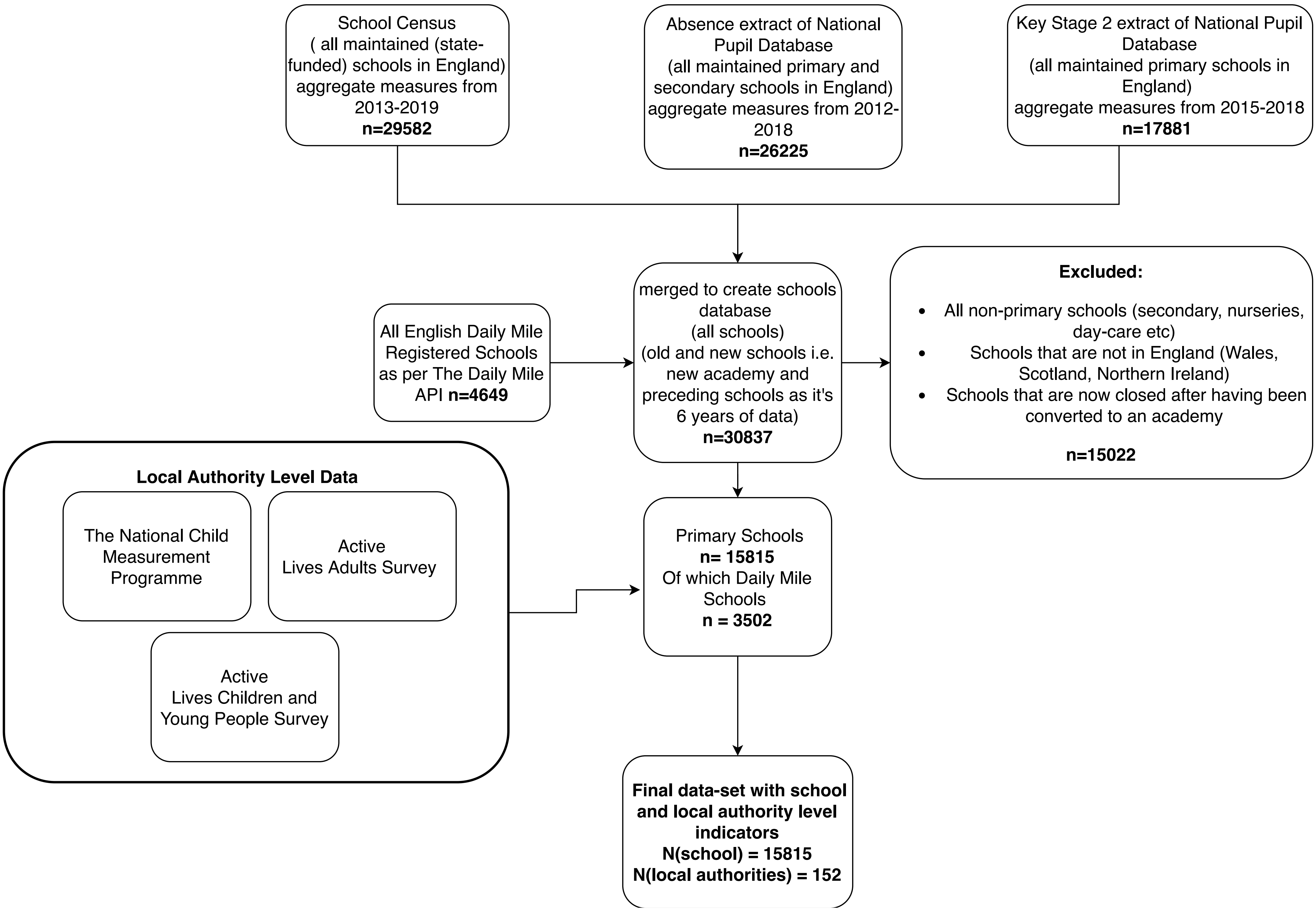

Supplement: Supplementary data [file jech-2020-214203supp001.pdf]
