# Supplementary material for: Sociodemographic profiles, educational attainment and physical activity associated with The Daily Mile™ registration in primary schools in England: a national cross-sectional linkage study
Source: J Epidemiol Community Health. 2020 Oct 1;75(2):137–44. doi: 10.1136/jech-2020-214203 (PMC7815899; doi:10.1136/jech-2020-214203)
Supplement: Supplementary data [file jech-2020-214203supp003.pdf]

SUPPLEMENTARY INFORMATION

Table S2: Multivariable logistic regression of school and local authority characteristics on The Daily Mile uptake in England using scaled parameters (N=15815 primary schools)

Parameters	Model 1b: intercept only		Model 2b: + school variables		Model 3b: + local authority variables	
	OR (95% CI)	Standard Error	Scaled OR (95% CI)	Standard Error	Scaled OR (95% CI)	Standard Error
**Local authority-controlled School**			1.03	0.05	1.03 (0.93, 1.13)	0.05
**Hamlets and Isolated Dwellings (Rural)¥**			0.91	0.12	0.94 (0.75, 1.18)	0.12
**Town and Fringe (Rural)¥**			0.94	0.08	0.96 (0.82, 1.12)	0.08
**Village (Rural)¥**			0.821*	0.08	0.84* (0.71, 0.98)	0.08
**Major Conurbation (Urban)¥**			1.67***	0.05	1.70*** (1.51, 1.91)	0.06
**Minor Conurbation (Urban) ¥**			1.13	0.12	1.02 (0.79, 1.33)	0.13
**% of pupils whose first language is known or believed to be other than English**			0.99	0.02	1.00 (0.95, 1.06)	0.03
**% of pupils reaching the expected standard in reading, writing, and maths**			1.00	0.02	1.01 (0.97, 1.06)	0.02
**% disadvantaged pupils**			1.01	0.003	1.01 (1.00, 1.01)	0.003
**% overweight or obese adults**					1.01 (0.94, 1.08)	0.04
**% physically active adults**					0.95 (0.89, 1.01)	0.03
**% physically active children**					1.00 (0.97, 1.05)	0.02
**% children who are overweight or obese**					0.93** (0.89, 0.98)	0.02
**Model Summary**						
**AIC**	16725		14508		12822	

*p≤0.05; **p≤0.01; ***p≤0.001

¥ (reference group = City and Town

AIC – Akaike Information Criterion
